# Direct Multi-Target Teaching Interface for Autonomous Handling of Multi-Stack Logistics in a Warehouse

**DOI:** 10.3390/s24175470

**Published:** 2024-08-23

**Authors:** Haegyeom Choi, Jaehyun Jeong, Taezoon Park, Donghun Lee

**Affiliations:** 1 Department of Mechanical Engineering, Soongsil University, Seoul 06978, Republic of Korea; choihg@soongsil.ac.kr; 2 Department of Chemical Engineering, Soongsil University, Seoul 06978, Republic of Korea; nfejjh@ssu.ac.kr; 3 Department of Industrial & Information Systems Engineering, Soongsil University, Seoul 06978, Republic of Korea; tzpark@ssu.ac.kr

**Keywords:** direct teaching interface, autonomous pick–place, target product recognition

## Abstract

This study presents a framework for enabling autonomous pick–place operations, addressing the need for efficiency in complex logistics environments using a direct multi-target teaching interface. First, tag and segmentation information were combined to recognize products in a complex warehouse, and a camera was installed on the rack to allow workers to remotely see the work environment, allowing workers to view the work environment in real time through a tablet. Workers can access the camera view showing the rack containing the target product through a swiping action and select the target product through direct teaching action. When the target product is finally selected, an optimal path is created through task planning, and an autonomous pick–place operation is performed based on the generated path. As a result of conducting a usability evaluation using the SUS (System Usability Scale) with six users on the interface that enables these tasks, it was confirmed that high user satisfaction was achieved with an average of 77.5 points. In conclusion, the proposed interface enhances operational efficiency and provides a user-friendly solution for complex warehouse tasks.

## 1. Introduction

**Figure 1:**
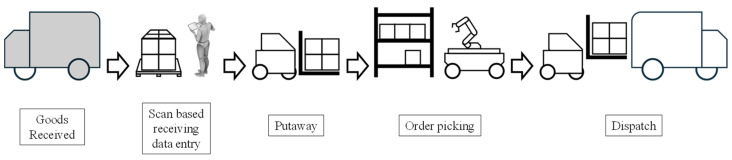
End-to-end process of the Warehouse Management System.

Global companies in traditional industries such as manufacturing and logistics are maximizing productivity and efficiency by applying and expanding robotics and automation technologies to solve productivity issues [1]. The pandemic has accelerated these efforts and led to explosive growth in the logistics market, necessitating the adoption of highly automated systems and advanced technologies. This surge in demand has driven the development of Logistics 4.0, defined as a logistics system that supports industrial and trade development by leveraging digital technologies to sustainably meet individualized customer demands without increasing costs in response to Industry 4.0 [2]. The Warehouse Management System (WMS), which manages logistics warehouses, is illustrated in Figure 1. Among the various WMS processes, order picking to fulfill customer orders stands out as the most costly, accounting for up to 55% of manual labor in logistics warehouses [3]. As a result, there has been a significant amount of active research and development focused on automating the order-picking process, with these efforts aiming to reduce manual labor, increase efficiency, and lower operational costs, ultimately enhancing the overall productivity of logistics operations.

**Figure 2:**
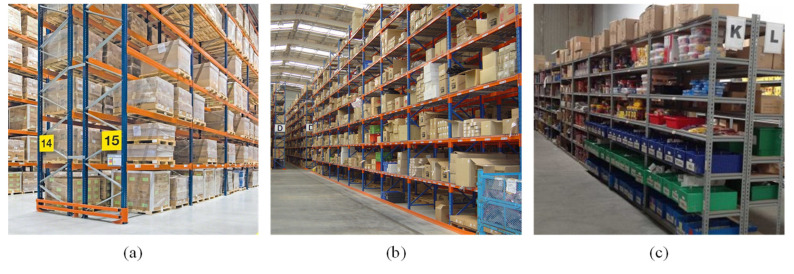
Results of classifying the warehouse into loading units: (**a**) the pallet-loading environment, (**b**) the box-loading environment, and (**c**) the piece-picking environment.

The automation of the order-picking process within a logistics warehouse can be classified into three environments based on the unit of loading, as shown in Figure 2: pallet-loading environment [4], box-loading environment [5], and piece-picking environment [6]. The pallet-loading environment is optimized for storing and transporting large quantities of identical products, making it suitable for large-scale distribution and handling large amounts of goods. The box-loading climate is ideal for managing various products efficiently and allows easy access to individual boxes when needed, making it suitable for handling diverse items. The piece-picking environment facilitates handling individual items and flexibly responds to customized orders, making it appropriate for e-commerce and retail industries. This study aims to automate the pick–place operations of target products in response to orders within the box-loading and piece-picking environments, which are conducive to handling various products and individual items.

Robotic systems capable of automating the order-picking process can be classified into forklift systems [7,8], grid-based systems [9], and mobile manipulator systems [10,11]. Forklift systems are used to move and load pallets on shelves within large spaces, making them commonly used in large logistics warehouses. Grid-based systems create a grid structure on the warehouse floor, allowing robots to move and retrieve items from designated locations efficiently. Lastly, mobile manipulator systems can perform various tasks, quickly adapt to changes in warehouse layouts, and handle small parts or products of different shapes. This study targets environments where multiple products and individual items must be managed. Therefore, instead of using forklift systems, which are suited for moving pallets in large warehouses, or grid-based systems, which are less flexible in dealing with product variations, this study focuses on utilizing mobile manipulator systems, which can flexibly handle products of different sizes and shapes.

In a typical logistics warehouse, there are methods to instruct the robot system to perform pick–place operations for the target product when an order comes in, such as a central management system [12,13,14] directly sending the product list, the quantity of each product, and the path to the rack where the products are located, directly to the robot. However, this approach requires the establishment of a WMS to manage inventory and location information, which incurs high initial costs and is effective in large, standardized warehouses that can afford these costs. On the other hand, in the unstructured environments targeted by this study, products change frequently and are a wide variety of sizes and shapes. Thus, the approximate location of the product can be known, but the exact pose is difficult to determine, so additional functions are needed for the automated system. Therefore, this study aims to enable autonomous pick-and-place operations by directly specifying the target product through a medium, calculating its pose, and conveying this information to the robot.

To instruct the target product through a medium-based direct teaching method, one can use a visual map reflecting the real-time logistics warehouse environment or directly teach by observing the work environment through the camera attached to the mobile manipulator. Speicher [15] designed and implemented an online shopping environment using a visual map to create an immersive virtual shopping environment where users can search and select items. However, digitalizing real-world items is very slow, so VR online stores may take some time to become practical and market-ready. Liang [16] pointed out that placing products on shelves using a mobile manipulator and pre-written programs is very difficult because the products in a store or warehouse are diverse and the layout changes frequently. Therefore, a human worker checked the graphical screen of the products on the shelf in real time using the interface and directly inputted the robot’s movements one by one to place the products on the shelf. In the study by Bormann [17], the target product can be taught remotely when the operator checks the camera view attached to the mobile manipulator in real time through RViz. However, if the target product is not visible in the current camera view, the robot is additionally controlled to make it visible.

Therefore, this study proposes a method to install rack-mounted cameras to ensure that the products on the racks in the work environment are visible, allowing workers to teach the target product remotely in real time through a tablet-based camera view. The tablet interface enables workers to sequentially check the camera views of each rack, facilitating an efficient search process. Workers can utilize a swiping action to switch different camera views, quickly locating the desired target product. Once the worker identifies and selects the target product through direct teaching actions on the tablet, the system calculates the precise location and pose of the target product. Using the calculated data, the system enables autonomous target product pick–place tasks, even in unstructured and dynamically changing environments.

The remainder of this paper is structured as follows: In Section 2, we present details about the proposed direct multi-target teaching method. Section 3 describes the results of the direct multi-target teaching method and the interface’s usability evaluation. Section 4 concludes this paper.

## 2. The Direct Multi-Target Teaching Method

### 2.1. Framework

**Figure 3:**
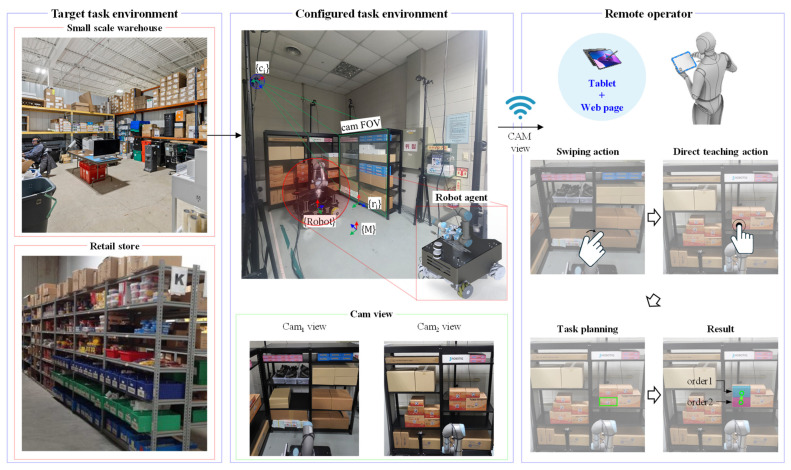
Overall framework for an autonomous target object picking system based on a direct multi-target teaching interface.

Figure 3 illustrates the framework of the direct multi-target teaching process and autonomous target product pick-and-place proposed in this study. The targeted work environments for this study are small-scale warehouses and retail shops, as shown on the left side of this figure. This study was conducted in a small-scale warehouse, and the robot agent used in the research was a mobile manipulator with omnidirectional mobility. In this work environment, rack-mounted RGB-D cameras were installed to face the opposite racks, ensuring comprehensive coverage and visibility of all products. The real-time footage from each camera is transmitted to the operator’s tablet via giga WiFi-6, providing high-speed and reliable data transfer. The operator can access the shelf video showing the target product through a swiping action on the tablet’s interface GUI, allowing them to switch between different camera views seamlessly. Using direct teaching actions within the real-time footage from a specific camera, the operator performs a multi-selection of target products. Once the final selection is complete, the precise poses of the target products are calculated and communicated to the robot agent. Subsequently, the robot agent performs indoor navigation to the target rack, recognizes the target rack, and executes multi-target product pick–place operations. Suppose other products are stacked on top of the target product. In that case, a task planning algorithm plans the optimal route to remove surrounding products sequentially, ensuring the structure of stacked products is maintained and the target product is picked efficiently.

### 2.2. Data Integration

**Figure 4:**
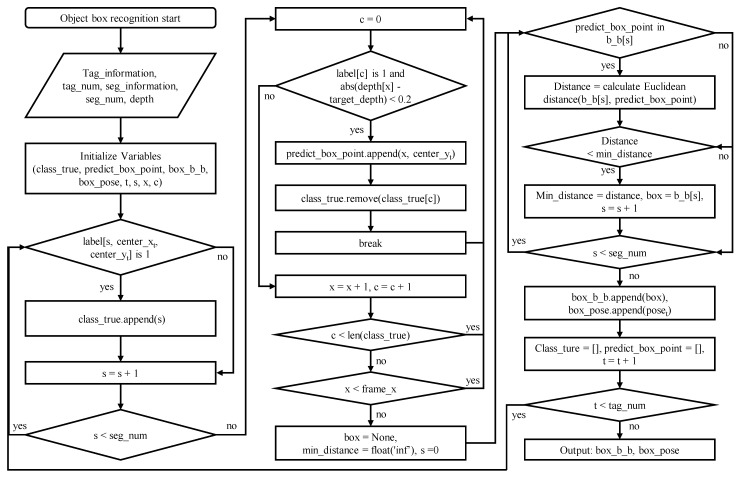
Flowchart for an algorithm that recognizes boxes by integrating tag information and segmentation information.

To recognize products and estimate the pose of the target product in a small-scale warehouse, it is first necessary to recognize the products on the racks. Since the products in a small-scale warehouse are boxes, image segmentation is needed to recognize each box, and instance segmentation must be used to identify each box containing different products. Among the various instance segmentation methods available, FastSAM and SAM-ViT-H are notable options. FastSAM [18] was used in this study due to its superior performance in real-time scenarios, making it well-suited for dynamic warehouse environments where quick and accurate segmentation is essential. FastSAM can effectively recognize all objects in the environment as individual instances. However, it cannot distinguish each instance, so each box cannot be recognized. To overcome this limitation, an additional step is required to combine the segmentation and tag information attached to each box. The system can accurately recognize each box and identify the product inside by integrating the tag information with the segmentation results obtained from FastSAM. The algorithm for this process involves several steps, including image acquisition, segmentation using FastSAM, and data integration with tag information. This algorithm’s detailed steps and logic are illustrated in the flowchart provided in Figure 4.

**Figure 5:**
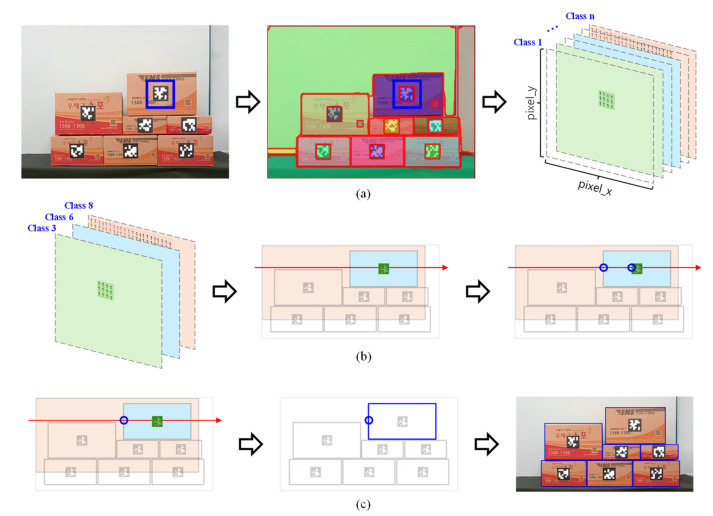
Process for recognizing boxes in a warehouse: (**a**) storing an instance of the tag center coordinates; (**b**) finding an instance of a box with a tag attached; and (**c**) storing a bounding box that matches the instance of the box among the bounding boxes.

To recognize only the product boxes in a small-scale warehouse, we first utilized FastSAM to input the recognized instance information, the tags attached to each box, and the depth information. Each box is tagged with a different ID to distinguish the product inside the boxes. Each instance with the center coordinates of each tag with a different ID indicates the presence of a box and tag, even if the specific label of the instance is unknown. Instances not including the box and tag may be background or other boxes located behind the primary box. All corresponding instances of tag center coordinates, including these instances, are stored, and this process corresponds to Figure 4 left and Figure 5a. Instances that are background or other boxes can be excluded using depth information, as they will be located behind the central coordinates of the tag. By comparing the depth information along the X-axis relative to the central coordinates of the tag, instances located further back can be excluded, leaving the coordinates where the tag and box instance start, which are then stored as shown in Figure 5b, corresponding to the center of Figure 4. Using the point that the tag is attached inside the box, the starting coordinates of the box’s instance can be selected, and the instance corresponding to the box with the attached tag can be identified. Finally, the target box can be recognized by choosing the bounding box closest to the box instance; this process is depicted in Figure 5c and corresponds to the right of Figure 4. By repeating these steps for each recognized tag, we can store the bounding box information of all boxes and the poses of the tags attached to them, allowing for each box’s recognition and pose estimation.

### 2.3. HMI and Direct Multi-Target Teaching Operation Design

**Figure 6:**
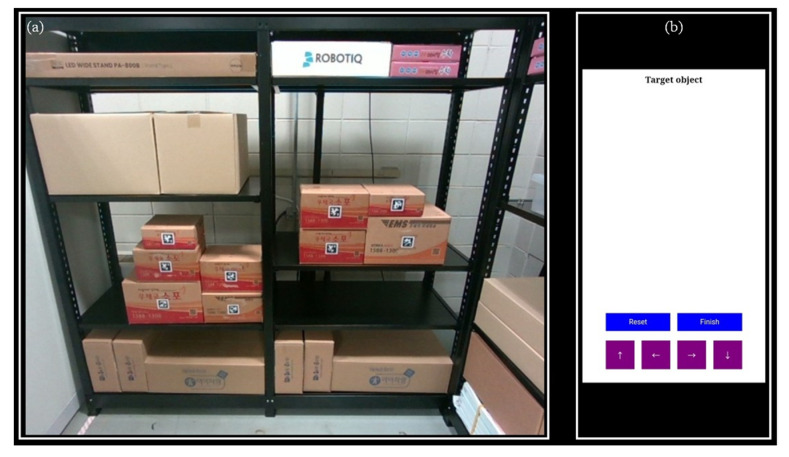
Direct multi-target teaching interface configuration for target product teaching: (**a**) the part where the camera’s view mounted on the rack in the work environment is shared in real time; (**b**) the part where the operator can check or modify the target product taught.

As shown in Figure 6, a direct multi-target teaching interface was configured to design a system where workers can instruct the ordered product to the mobile manipulator system. HTML was used to create a web page for the direct multi-target teaching interface, and for security reasons, the tablet PC can only access the interface when it is on the same network as the work environment. The main functions of this interface are as follows: (1) sharing the rack-mounted camera view with the remote worker; (2) allowing the worker to access the camera showing the target product through a swiping action; and (3) enabling the selection of the target product on the shelf through direct teaching actions.

**Figure 7:**
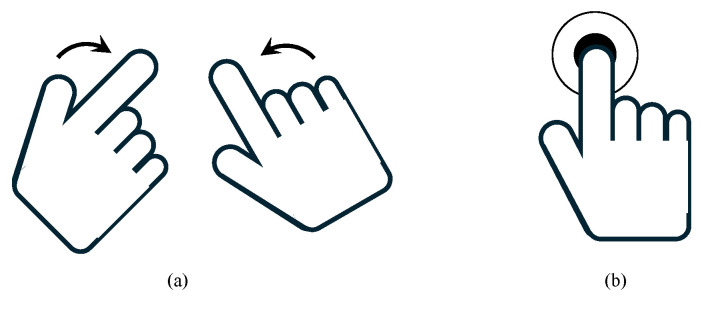
Action definitions for controlling the direct multi-target interface: (**a**) swiping action to access the camera viewing the rack with the target product; (**b**) direct teaching action to teach the target product.

The rack-mounted camera view in the work environment is linked to the tablet, and one of the linked camera views is shared in real time, as shown in Figure 6a, to check for the presence of the target product on the rack. Suppose the target product is not on the rack or visible in the current camera view. In that case, the operator can use a swiping action, as seen in Figure 7a, to switch to another camera view showing different racks to locate the target product. Once the target product is found, the operator selects it using direct teaching action, as illustrated in Figure 7b. The selected target product is edge-highlighted, and a list of the target products can be viewed in Figure 6b. If the wrong target product is selected, it can be removed using the reset button. The autonomous pick–place operation begins when the worker has finished selecting the target product and clicks the finish button.

### 2.4. Task-Planning

**Figure 8:**
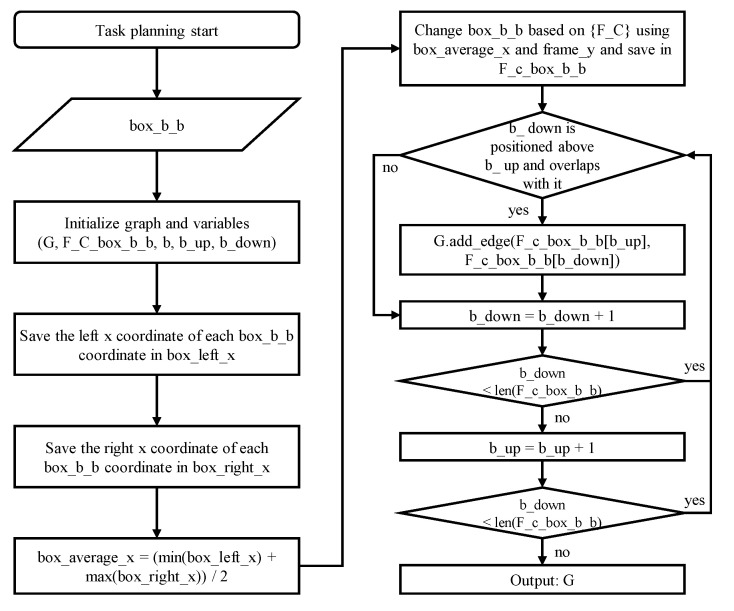
Flowchart of the task planning algorithm for picking target objects by the shortest path.

After selecting the target products through the direct multi-target teaching interface, it is essential to ensure that the stacked box structure does not collapse during autonomous target product picking. To stably remove the target product like this, each recognized product is created as a node, and an optimal path is planned to minimize the time required through task planning that connects the nodes to edges by considering the positional relationship. The target product is picked by sequentially removing products surrounding the target product according to the created path, and the task planning algorithm used at this time is shown in Figure 8.

**Figure 9:**
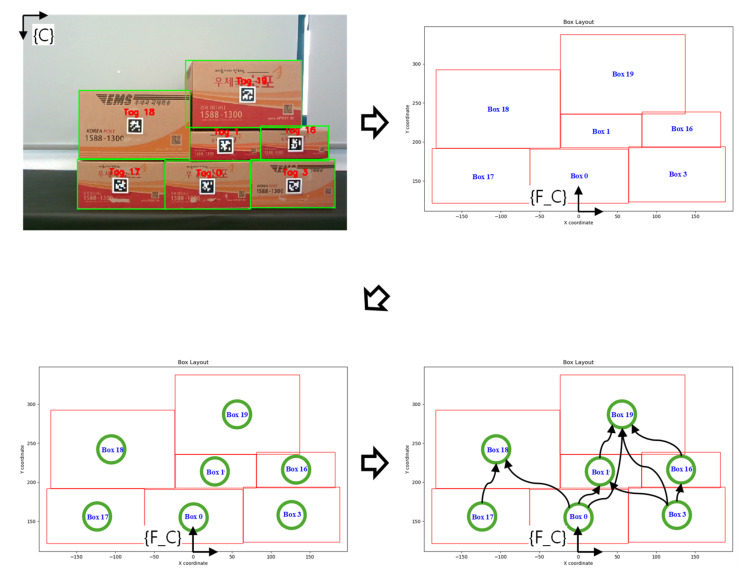
The task planning process that determines the optimal pick–place order through the shortest path.

Task planning is performed by receiving the box’s bounding box as input, resulting from data integration combining tag and segmentation information. However, since the bounding box coordinates are obtained based on the camera frame, where the upper left is the reference point, and the Y-axis increases downward, the reference coordinate system is changed to {F_C} as shown in Figure 9, corresponding to the left side of Figure 8. The bounding boxes of the products based on the changed coordinate system are created as each node. The upper-level box must be subtracted first to subtract the lower-level boxes, so check whether the upper-level box exists based on each product. If an upper box exists, there are two cases: the upper box and the lower box either overlap horizontally or do not overlap. If they do not overlap, the upper box does not need to be removed to remove the lower box, so this case is skipped. If they overlap, the upper box must be removed to remove the lower box. Therefore, in this case, the upper box and lower box nodes are connected by edges, creating a configuration shown in Figure 9, corresponding to the right side of Figure 8.

Once the worker selects the target product as input, the optimal path for extracting the target chosen product from the stacked structure is generated through task planning using graph G. The pose of the product corresponding to the optimal path can be known based on the camera, and since the camera is installed on the rack, the pose of the product can be converted based on the rack. Subsequently, knowing the position of the rack and the mobile manipulator on the map, the product’s pose relative to the camera can be converted to the mobile manipulator’s frame using Equation (1). Afterward, autonomous target product pick–place can be performed through the product’s pose based on the mobile manipulator.

(1) Ttrobot=TMrobot·TriM·Tciri·Ttci

## 3. Experiment and Results

### 3.1. Object Box Recognition and Task-Planning

**Figure 10:**
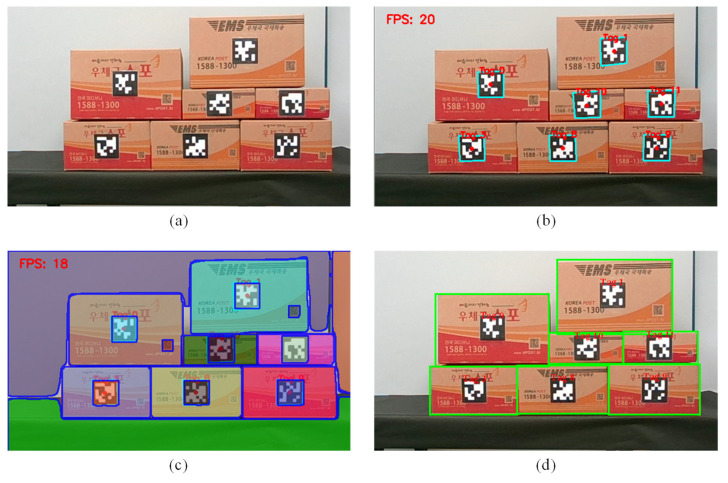
Results of recognizing a box as a product through data integration: (**a**) original image; (**b**) tag recognition result; (**c**) segmentation result using FastSAM; (**d**) final result of box recognition by integrating tag and segmentation information.

Racks were installed to construct an environment similar to the small-scale warehouse; boxes with tags on the rack were stacked to create an atmosphere; and the automatic target product pick–place proposed by this study was conducted within the environment. First, to recognize only the boxes, which are the products on the racks, the result of data integration combining tag information and segmentation information can be seen in Figure 10. The original image from the rack-mounted camera is shown in Figure 10a; the result of tag recognition can be seen in Figure 10b; and the result of segmentation using FastSAM is shown in Figure 10c. The result of recognizing only the boxes through data integration using tag recognition and segmentation information is displayed in Figure 10d. Through these results, it can be confirmed that the boxes were accurately recognized and that the location of each box can be saved using tag information.

**Figure 11:**
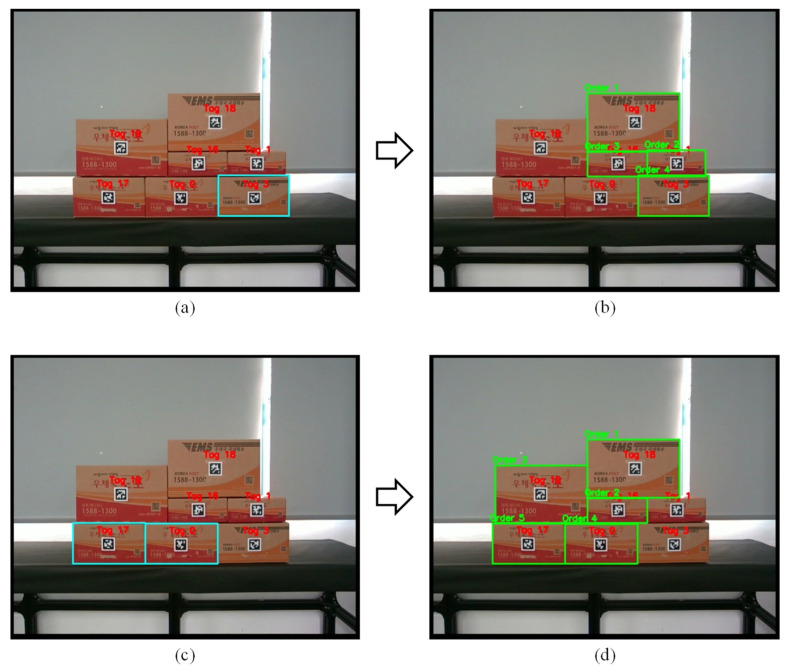
Shortest path creation results for target product picking: (**a**) result of selecting one target product; (**b**) shortest path result with one target product; (**c**) result of selecting multi-target products; (**d**) Shortest path result with multi-target products.

Task planning was performed through the bounding box of the recognized boxes to create each recognized box as a node, and a path to subtract target products from the shortest route was created when target products were given by connecting nodes to edges according to location relations. First, as shown in Figure 11a, edge highlighting occurs when the operator remotely selects the box at the bottom right as the target product through direct multi-target teaching. To pick the target product, examining the positional relationships reveals that two boxes are directly above the target product, and one box is above these two boxes. Therefore, picking the target product requires first picking the three upper boxes, as shown in Figure 11b. Next, when the worker selects the two lowermost target products, as shown in Figure 11c, the shortest path planning result for picking the two target products can be seen in Figure 11d. This demonstrates that the worker can remotely select target objects through direct multi-target teaching actions and generate the shortest path for autonomous pick-and-place operations.

**Figure 12:**
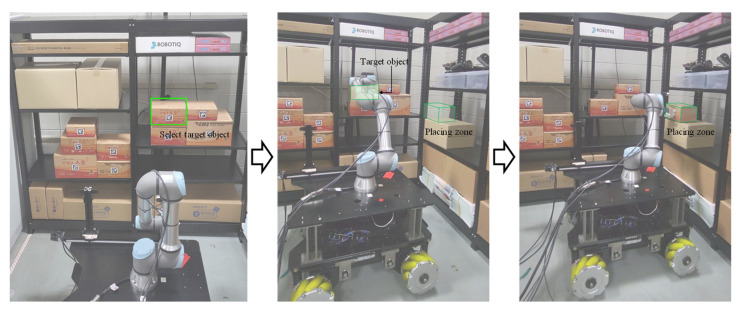
Results of autonomous target product pick–place within the work environment.

The task planning algorithm generates the optimal path that minimizes the required time for selecting the target products through direct teaching actions based on the direct multi-target teaching interface. Figure 12 shows the result of performing the target product pick–place by converting the pose of the corresponding product into the mobile manipulator’s frame. We used Universal’s ur5e as a manipulator to produce a mobile manipulator. We designed an omnidirectional mobile robot by dividing the mobile into driving, controller, and sensor levels to move freely within a complex and dynamic work environment. ROS-based MoveIt and indoor navigation were used to control the corresponding mobile manipulator. MoveIt was utilized for the manipulator’s motion planning, and indoor navigation was implemented for autonomous driving of the mobile base.

### 3.2. Interface Usability Evaluation

The System Usability Scale (SUS), which can evaluate usability on hardware, software, websites, and applications, was used to assess the direct multi-target learning interface. The SUS scores ten items by selecting one of five responses ranging from “very disagree (1 point)” to “very agree (5 points)”, and the higher the score out of 100, the higher the satisfaction with the interface. Additionally, according to Jeff Sauro’s research [19], the average SUS score for evaluating user interfaces is 68. A score above 80.3 suggests that users will likely recommend the interface to others.

**Figure 13:**
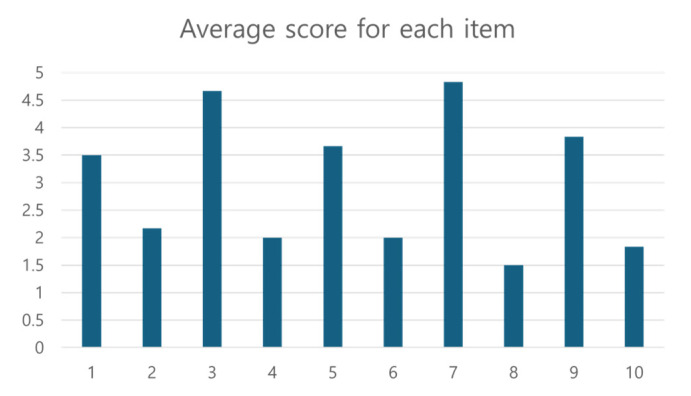
The average score for each item among the results of usability evaluation using the SUS for multi-target education interface by six users.

The direct multi-target learning interface’s usability evaluation was conducted with six users. Figure 13 shows the score and average for each user, and Table 1 shows the average score for each item. Given that the average SUS score is 68, the average SUS score of 77.5 for the interface proposed in this study indicates that it is more user-friendly than average. Among the items, odd-numbered items indicate positive responses when scored higher, while even-numbered items indicate positive responses when scored lower. The scores for items 1 and 2 are relatively lower than others. Item 1 pertains to how frequently the user would use the interface, and item 2 relates to the complexity of the interface, suggesting that the interface’s complexity may make frequent use challenging. Upon asking users about the complexity, it was found that the swiping action required to access the rack-mounted camera view and see the target product was considered repetitive and contributed to the lower scores. Conversely, items 7 and 8 received the highest scores. Item 7 concerns the time required to learn how to use the interface, and item 8 concerns the discomfort of using the interface. Therefore, it means that the interface used in this study is convenient.

## 4. Conclusions

This study introduces a novel framework that enables workers to remotely access rack-mounted cameras, view or select target products using HMI-based direct multi-target teaching actions, and execute autonomous pick-and-place operations along the shortest path. A work environment and a logistics warehouse were set up to validate the proposed method. The worker used the HMI-based interface to remotely select target products and perform autonomous pick-and-place operations, leading to the following results:

1 Box recognition in the warehouse: Only the box can be accurately recognized within the complex logistics warehouse by combining the box’s attached tag information with the segmentation result.
2 Shortest path for target product pick–place: By creating nodes from the recognized boxes and connecting each node with edges based on the positional relationships of the boxes, it is possible to generate the shortest path and perform autonomous target product pick–place operations regardless of the number of target products.
3 Direct teaching interface usability evaluation: The interface used to perform the above tasks remotely received an average SUS score of 77.5, indicating a high level of usability.

The above results demonstrate effective box recognition and remote product selection via the interface, facilitating efficient task execution along the shortest path. However, there is an issue with the interface requiring repetitive swiping actions to access the camera view where the target product is visible if it is not currently in view. Furthermore, the task planning algorithm can determine the optimal order first by selecting the top box to prevent the structure from collapsing if it comes within the camera FOV, regardless of the size of the structure. Still, there is a limitation: it always selects the top box first, even when the structure does not collapse and the target product can be chosen directly, just like playing Jenga. Therefore, future work will involve designing an interface that allows viewing and selecting from all camera views simultaneously, eliminating the need for repetitive swipe actions. Additionally, a network capable of assessing structural stability will be developed to determine whether the target product can be picked directly from the stacked structure without causing it to collapse, thereby overcoming the mentioned limitations.

## Figures and Tables

**Table 1:** The score and average for each user among the results of usability evaluation using the SUS for multi-target education interface by six users.

User	System Usability Scale Score
U01	65
U02	80
U03	80
U04	85
U05	75
U06	80
Average	77.5

## Data Availability

The original contributions presented in the study are included in the article, further inquiries can be directed to the corresponding authors.
